# Lady Physicians

**Published:** 1883-04

**Authors:** E. F. Ingals


### ARTICLE VI.

### LADY PHYSICIANS.

At the banquet of The Alumni Association of the Women's Medical College, Prof. E. Fletcher Ingals responded to the toast, "*The Profession*," in the following strain, which will represent the status of lady physicians in the West:

Within a few years, there has been a great change in the feeling of the profession toward lady physicians. This is nowhere more clearly shown than by action of different medical societies of this city, of the Illinois State Medical Society, and of the American Medical Association, all of which have admitted ladies to full membership.

This is due in part to the ladies themselves, but also to the institutions from which they are graduated.

A few years ago, women, with the peculiarities of Dr. Mary Walker, were attempting to crowd themselves, not only into the profession of medicine, but into all of the professions and arts, where formerly only men had been employed. This was done at the expense of those traits which have always been considered as the peculiar glory of womanhood; and, as a result, gentlemen in and out of the profession, saw in the effort only a tendency to the degradation of the gentler sex, and many would rather have followed their sisters to the grave, than to have seen them so associated with these strong-minded women. But, the ladies, who are active in the profession and who are now studying, do not countenance these methods, and hence a great change has come; however, much of this change, particularly in the West, is due to the institution in whose honor we are convened here to-night.

However great the objections may be to the study of medicine by ladies in mixed classes, and I, for one, think these objections have not been overrated, there can be no possible objection to its study in institutions like the college you call Alma Mater.

In entering this institution, a lady sacrifices nothing of her delicacy, modesty or relf-respect, but takes a position parallel with that of her brother in any of the best colleges of the country.

The profession and the public appreciate this, and gladly extend the hand to the lady physician, which a few years since they would have withheld from the *female doctor.*

I predict, that when the objectionable features, which still remain in our larger hospitals, can be removed; when the fact, which was long ago accepted by the foremost colleges of the country, and which has been recently acted upon by the more advanced Homœopathic colleges—namely, that mixed classes are not good, shall be universally accepted, then the last lingering prejudices will disappear, and, then, ladies will take an equal position with their brothers in the great work of ministering to the sick.

The profession already welcome the graduates of this college, as they do gentlemen from other institutions of high grade; but it is still necessary for a lady physician to tell where she graduated, when she receives an introduction.

It is often urged that lady physicians cannot endure the hardships, which must be borne by general practitioners, and this is probably true, but this is only one of the misfortunes which will render your success the more conspicuous.

However, the profession is broad, and those who find themselves unable to endure the hardships of general practice, may after a few years settle into some of the specialties into which the practice of medicine is divided. But none, I hope, will attempt to practice any of the ordinary specialties, until they have had several years experience in general practice, for without such an experience no one is competent to enter a special field, unless I except two of the specialties which are now taught in this institution.

I refer to Pharmacy and Dentistry. The former is not recognized as a specialty of medicine, but it should be, and there is nothing about it which the competent lady physician cannot attend to.

The latter has but recently been acknowledged as one of the proper specialties of medicine. It opens a large and profitable field for usefulness for any of our graduates, who have not the physical ability to endure general practice, or who prefer to concentrate their work upon a single line, in order that they may do that the better.

In conclusion, placing myself, for the moment, outside of the Faculty and speaking for medical men at large, I welcome the graduates of this college, and of similar institutions, to all the honors and emoluments of the profession.

